# Supplementary material for: Incidence of common infectious diseases in Japan during the COVID-19 pandemic
Source: PLoS One. 2022 Jan 12;17(1):e0261332. doi: 10.1371/journal.pone.0261332 (PMC8754328; doi:10.1371/journal.pone.0261332)
Supplement: S1 Fig — S1A: Cross-correlation between COVID-19, and common infectious diseases under the national sentinel surveillance. S1B: Cross-correlation between COVID-19, and common infectious diseases under the national notifiable disease surveillance. The X-axis indicates the correlation coefficient between COVID-19 and each common infectious disease. The Y-axis indicates lag (week). (PPTX) [file pone.0261332.s001.pptx]

## Slide 1
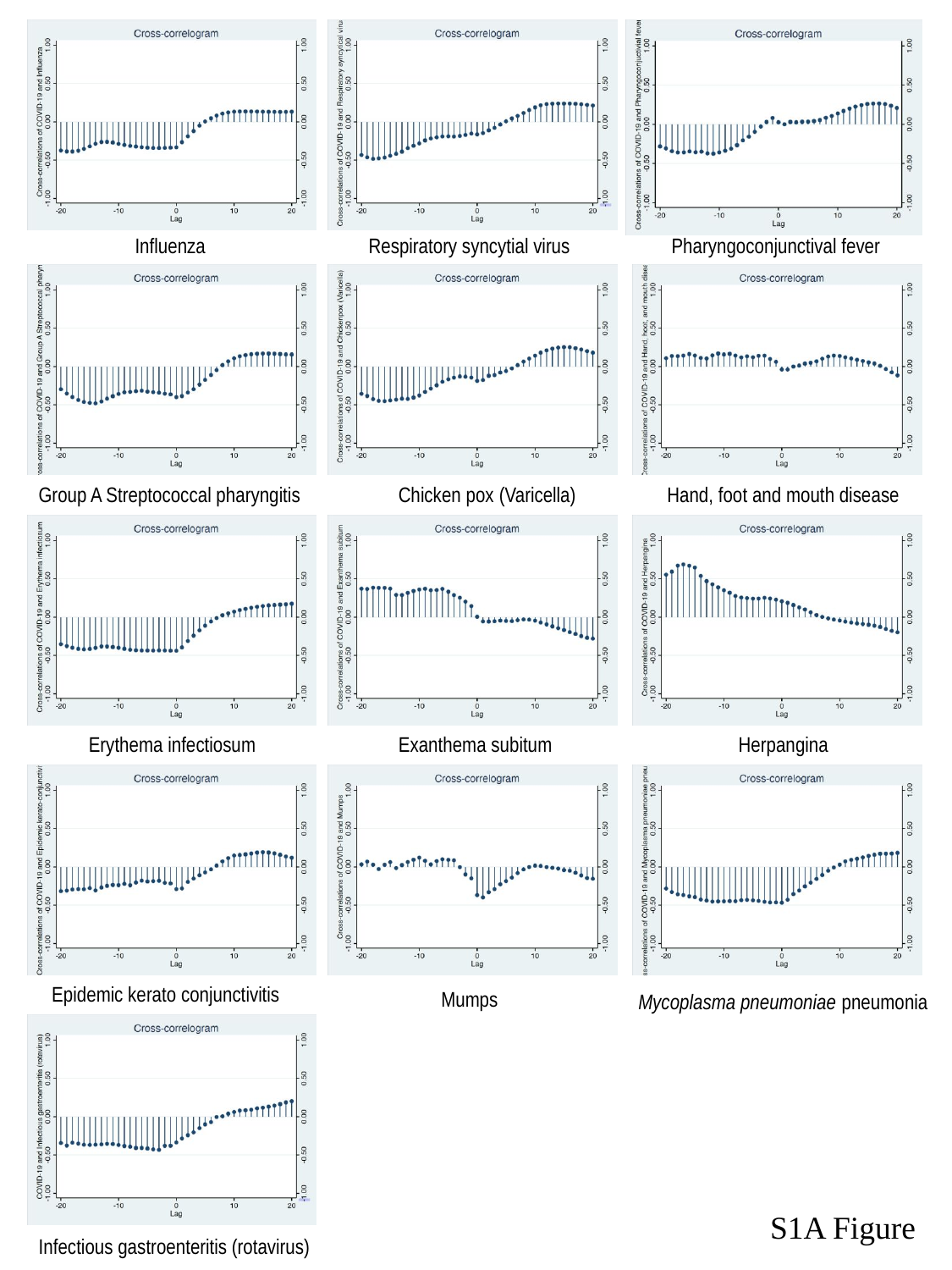

Influenza
Respiratory syncytial virus
Pharyngoconjunctival fever
Group A Streptococcal pharyngitis
Chicken pox (Varicella)
Hand, foot and mouth disease
Erythema infectiosum
Exanthema subitum
Herpangina
Epidemic kerato conjunctivitis
Mumps
Mycoplasma pneumoniae pneumonia
S1A Figure
Infectious gastroenteritis (rotavirus)

## Slide 2
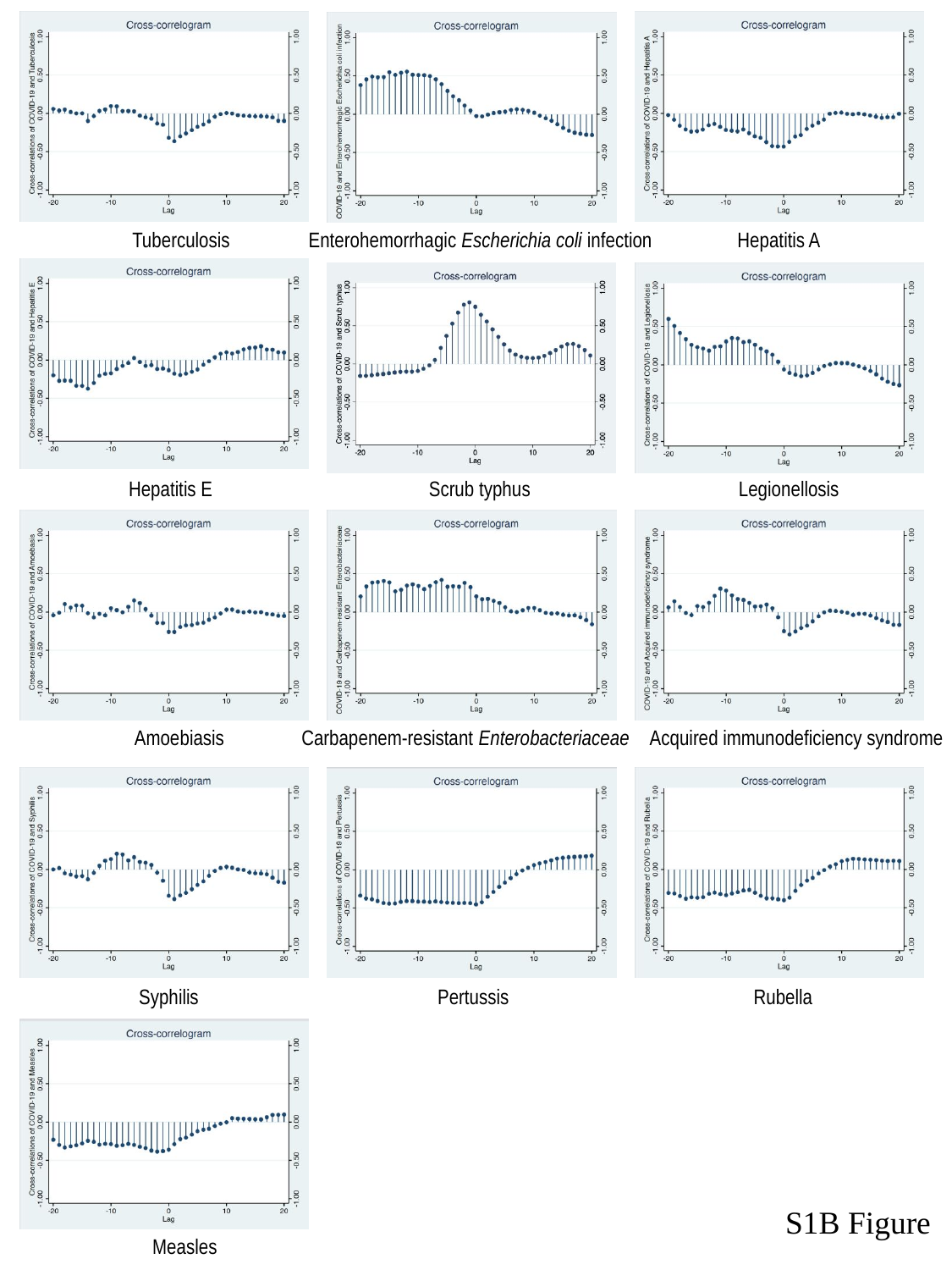

Tuberculosis
Enterohemorrhagic Escherichia coli infection
Hepatitis A
Hepatitis E
Scrub typhus
Legionellosis
Amoebiasis
Carbapenem-resistant Enterobacteriaceae
Acquired immunodeficiency syndrome
Syphilis
Pertussis
Rubella
S1B Figure
Measles

## Slide 3
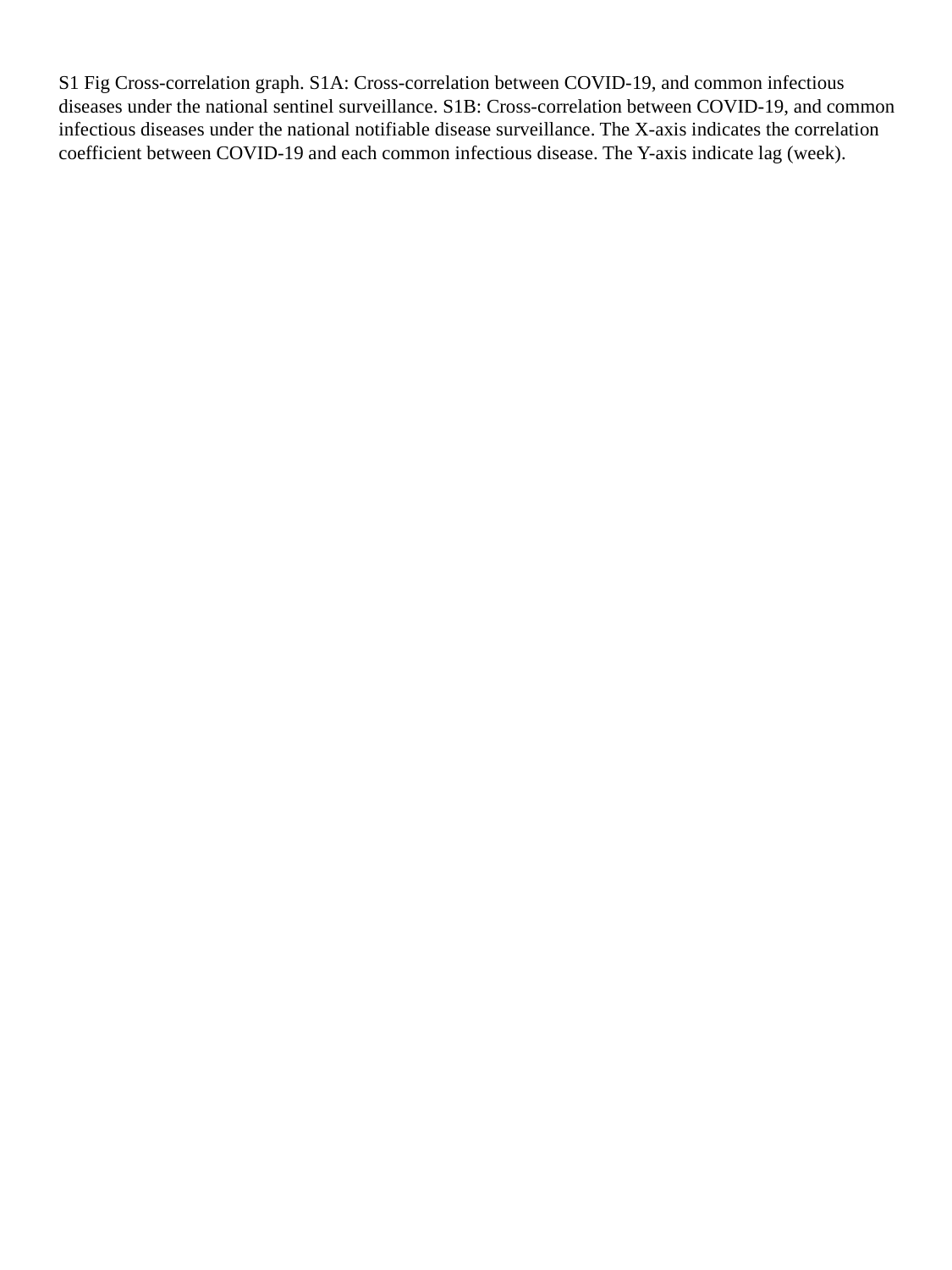

S1 Fig Cross-correlation graph. S1A: Cross-correlation between COVID-19, and common infectious diseases under the national sentinel surveillance. S1B: Cross-correlation between COVID-19, and common infectious diseases under the national notifiable disease surveillance. The X-axis indicates the correlation coefficient between COVID-19 and each common infectious disease. The Y-axis indicate lag (week).
