# Supplementary material for: Incidence of common infectious diseases in Japan during the COVID-19 pandemic
Source: PLoS One. 2022 Jan 12;17(1):e0261332. doi: 10.1371/journal.pone.0261332 (PMC8754328; doi:10.1371/journal.pone.0261332)
Supplement: Attachment — Submitted filename: Response_to_Reviewers.docx [file pone.0261332.s004.docx]

5. Review Comments to the Author

Reviewer #1: My comments (in no particular order) are focused primarily on the authors’ methods and presentation.

1. The graphs indicate “week” along the x-axis. Does this represent calendar week (i.e., starting from January 1) or “seasonal” time (e.g., one might take influenza season as extending from July to June).

Reply to the reviewer: The week on the horizontal axis of Figure 2 and Figure 3 indicates an epidemiological week (for example, the first week in 2020 is from December 30 to January 5, and the first week in 2019 is from December 31 to January 6). The definition of weeks was based on the “Report week correspondence table” (see <https://www.niid.go.jp/niid/ja/calendar.html>) presented by the National Institute of Infectious Diseases. Although the definition of epidemiological weeks varies from year to year, but the difference is only a few days, not large enough to affect over the months.

We added the explanation about the “week” in the main text.

**Material and Methods** (inserted on page 8, line 125-129)

Though the actual reported numbers were reported per week, this “week” indicates an epidemiological week. For example, the first week in 2020 is from December 30 to January 5, and the first week in 2019 is from December 31 to January 6. This is referred to as the “weeks ending log” prescribed by the National Institute of Infectious Diseases (see https://www.niid.go.jp/niid/en/calendar-e.html).

**Description of Fig 2** (inserted on page 14, line 211)

The “week” along the x-axis indicates the epidemiological week.

2. It is not at all clear to me how one can observe fractional numbers of cases per week

(e.g., as with respiratory syncytial virus).

3. The authors present counts from (A) national sentinel surveillance (e.g., Figure 2A) and (B) national notifiable disease (e.g., Figure 2B). Since numbers are much smaller for the former versus the latter, the question arises how well A tracks B. Are there any common diseases to A and B, and if so, are the patterns similar?

Reply to the reviewer: We do apologize for the confusion, as the fractional numbers of cases per week were unique to Japan and were difficult to understand. In Japan, fractional numbers of cases per week surveys are conducted for common diseases, and 100% of surveys are conducted for important diseases for which it is judged how all cases should be grasped. Figure 2A shows survey results of the fractional numbers of cases per week. Table 1 shows the number of facilities for each fractional numbers of cases per week. The vertical axis is the number of cases diagnosed at each facility per week divided by the number of facilities. Since it is difficult to understand the expression method on the vertical axis, we will change it to the expression method we have used in our previous articles as follows: The vertical bar represents the number of patients referred to one hospital per week per selected time point. On the other hand, Figure 2B shows a 100% survey. Since the fractional number of cases per week survey or the 100% survey is decided depending on the disease, these data cannot be duplicated.

**Material and Methods** (inserted on page 6, line 103-115)

Sentinel surveillance systems involve clinics or hospitals or public health centers, local infectious disease surveillance centers (local IDSCs), and national infectious disease surveillance centers (national IDSC). The public health center gathered a total number of patients during one week with target diseases diagnosed at each medical facility with influenza, pediatric, ophthalmic, and designated sentinel sites. Local IDSCs gather the data from public health centers in a prefecture. Finally, national IDSC gather the data from local IDSCs. The weekly number of cases includes number of cases diagnosed at each facility per week divided by the number of facilities with sentinel sites. The weekly number of reports is calculated by dividing the weekly number of patient reports by the number of medical institutions with sentinel sites. This number of sentinel sites is to reflect Japan’s overall trends regarding infectious disease epidemics. Sentinel sites are set under the jurisdiction of the public health center so that the morbidity rate in nationwide can be estimated with a standard error rate of ≤5%.

**Legends of Table 2** (inserted on page 11, line 146-147)

The vertical bar represents the number of patients referred to one medical facility per week per selected time point, but italic figures show the total number by 100% survey.

**Figure legends** (inserted on page 14, line 204-205)

Each left vertical axis represents the number of patients referred to one medical facility per week per selected time point (a) or the total number of cases per week reported (b).

4. The authors might formalize the relationship (if any) between disease counts and COVID-19 incidence by examining the cross correlation function between the two time series. It would be of interest if lags could then be identified.

Reply to the reviewer: We examined the cross-correlation function between the two-time series of COVID-19 and common infectious diseases count and described it in the Results section. In addition, cross-correlogram of each disease are shown as supplemental figures. We added statistics analysis in the Material and Methods section as follows:

**Material and Method**

**Statistical analysis** (inserted on page 12, line 158-169)

We examined the cross-correlation functions (CCF) between the two-time series of common infectious disease count and COVID-19 disease count. We used the CCF to understand the time-lagged correlation between common infectious disease count and COVID-19 incidence. To compare the time series change in case numbers of common infectious diseases in 2020 with that in the previous 5 years (2015–2019), a difference-in-differences linear regression was applied for infectious diseases transmitted by droplets that are most susceptible to behavioral changes caused by COVID-19. The model included a categorical variable for each week, a categorical variable for the 2020 season (versus the 2015–2019 seasons) and the interaction variables for each week and the 2020 season, following the method described by Sakamoto et al. (2020) [5]. For the statistical analysis, Light Stone^®^ STATA^®^ ver.15 was used.

**Results** (inserted on page 17, line 255-259)

Additionally, we examined the CCF between the two-time series of COVID-19 and common infectious disease counts. Overall, there were no diseases showing a strong correlation with COVID-19 (S1 Fig). Among them, cross-correlations in Scrub typhus were the highest at rag –1 week (CCF 0.8075), and cross-correlations in herpangina were the highest at rag –15 week (CCF 0.6465).

5. One might more readily be able to identify trends or periodicities in the graphs if the authors were to smooth the incidence curves (e.g., moving averages). Given counts from national sentinel surveillance are typically quite small (in the single digits), eliminating to some extent random fluctuations might be useful.

Reply to the reviewer: Thank you for your advice. The moving average lines have been added to the column graph of COVID-19 and the incidence curve of common infectious disease. We append this to the section of Materials and Methods as follows;

**Material and Methods**

**Figure legend** (inserted on page 14, line 208-211)

The moving average lines were applied to the column graph of COVID-19 incidence (gray tick line) and to the incidence curve of 2020 (scarlet tick line) of each common infectious disease.

**Figure legend** (inserted on page 17, line 268-270)

The moving average lines were applied to the incidence curve of 2020 (scarlet tick line) and 2015-2019 (blue tick line) of each common infectious disease.

6. In a similar spirit, would it be useful to combine diseases in Figure 3 according to mode of transmission? Would a clearer pattern thereby emerge?

Reply to the reviewer: Thank you very much for your comment that each disease in Figure 3 should be combined. However, if you look closely at Figure 3, there is a big difference for each disease, especially for oral infections. As suggested by the reviewer, we also think that it is important to consider each infection route. However, since there are differences for each disease, and we believe that it is more accurate to show individual data.

7. Why counts and not rates? And, should not there be age adjustments, or gender considerations? For example, the “usual” U-shaped incidence curve for influenza is lost by pooling across all ages.

Reply to the reviewer: Thank you for your important suggestions. We have already responded to Japan's unique surveys on fractional numbers of cases per week. As for the influenza that you pointed out, as shown in Figure 2, since the number of definitive diagnoses continues to be extremely small in 2020, it is impossible to consider age and gender. For this reason, we judge that it is important to understand the overall picture of influenza rather than considering age and gender. For respiratory syncytial virus infections, it is especially important to consider age, and there are limited epidemics in some regions in Japan in 2021. Regarding respiratory syncytial virus infections, the authors would like to perform the analysis considering age and gender in future studies.

**Discussion**

**Limitation of this study** (inserted on page 33-34, line 603-612)

It will be necessary to adjust age and gender for the incidence of each infectious disease [69]. However, as for the influenza shown in Figure 2, since the number of definitive diagnoses continues to be extremely small in 2020, it is impossible to consider age and gender. For this reason, we judge that it is important to understand the overall picture of each disease rather than considering age and gender. For respiratory syncytial virus infections, it is especially important to consider age, and there are limited epidemics in some regions in Japan in 2021 [70]. Regarding respiratory syncytial virus infection, the authors would like to perform the analysis considering age and gender in future studies.

**References** (added new citation: page46-47, line850-856)

71. Galasso V, Pons V, Profeta P, Becher M, Brouard S, Foucault M. Gender differences in COVID-19 attitudes and behavior: Panel evidence from eight countries. Proc Natl Acad Sci U S A. 2020;117: 27285-27291.

72. Ujiie M, Tsuzuki S, Nakamoto T, Iwamoto N. Resurgence of Respiratory Syncytial Virus Infections during COVID-19 Pandemic, Tokyo, Japan. Emerg Infect Dis. 2021;27.

8. My revision

We needed a reference for the statistical analysis in this revision. Therefore, I added two authors for this paper.

Hiroyoshi Iwata, Clinical Pharmacology & Therapeutics, University of the Ryukyus School of Medicine

Shinichiro Ueda, Clinical Pharmacology & Therapeutics, University of the Ryukyus School of Medicine

We changed “Varicella” to “Chicken pox” because the name of “Chicken pox” is more common in children. Similarly, pertussis is sometimes used as the name of “Whooping cough;” thus, it is shown in parentheses.

In this revision, we added supplemental table and figure. Therefore, I described the supporting information.

**Supporting information** (inserted on page 47-48, line 858-878)

**S1 Fig. Cross-correlation graph.** S1A: Cross-correlation between COVID-19, and common　infectious diseases under the national sentinel surveillance. S1B: Cross-correlation between COVID-19, and common infectious diseases under the national notifiable disease surveillance. The X-axis indicates the correlation coefficient between COVID-19 and each common infectious disease. The Y-axis indicates Lag (week).

**S1 Table. Case numbers per week about COVID-19 and case number per week per sentinel cite of notifiable infectious diseases.** These weekly numbers of COVID-19 were calculated from the daily number of the new positive cases that have taken polymerase chain reaction for SARS-CoV-2 or antigen testing for SARS-CoV2. The data was obtained from the Ministry of Health, Labour and Welfare [7]. The numbers of notifiable infectious diseases were collected from the Infectious Diseases Weekly Reports in accordance with the National Epidemiological Surveillance of Infectious disease [8].

**S2 Table. The total number of out-of-hospital (outpatient and home medical care) cases in the medical clinics and insurance pharmacies compared with that in the same month of the previous year.** The number of cases referred to here is the number of medical fee statements (receipts), and each medical institution prepares one statement for one patient every month. The data is national data and are obtained from the Ministry of Health, Labor and Welfare's estimated medical expenses database [11].

Reviewer #2: In this manuscript, the authors aim to evaluate whether restrictions and interventions in place during COVID-19 in Japan were associated with decreases in reported cases of other infectious diseases. They compare total case counts between the years 2019 and 2020, as well as epidemic curves for weekly cases, and find that several pathogens, particularly those transmitted by the droplet route, had strikingly lower numbers of reported cases during the COVID-19 epidemic in 2020. They also include a very thorough discussion of their results for various pathogens, including results that were unexpected, which I appreciated.

Overall, the comparison of epidemic curves, and total cases, between 2019 and 2020 support the overall conclusion that the measures taken to curb the spread of COVID-19 may have also prevented the spread of certain infections. My main comments here are (1) whether a statistical analysis can be used to quantify weekly changes in case counts as well as to tie the story together a bit more clearly, and (2) how much of the reduction in these other infections might be attributable to reduced healthcare seeking and/or reduced capacity to test for other pathogens during COVID-19. Please find these and additional comments described in detail below.

Main comments:

1) I suggest using a difference-in-difference regression model to statistically evaluate changes in weekly case counts for each of these pathogens. While I very much appreciate the detailed discussion of the trends for each pathogen, and I agree that the data speaks for itself in many of the examples, I think a quantitative analysis such as this would help pull the manuscript together with a clearer story. This would also help to identify which week(s) during 2020 each pathogen had significantly different case counts from the previous year(s). Examples of this approach can be found in the Sakamoto et al. (2020) JAMA analysis of changes in influenza epidemics in Japan, as well as in the Lee & Lin (2020) EID study on effects of COVID-19 on infections in Taiwan.

Reply to the reviewer: We applied to our data a difference-in-differences regression model to statistically evaluate change in weekly case counts for each of the infectious diseases. We then described the statistical analysis in the Material and Methods and Results sections as follows:

**Material and Methods**

**Statistical analysis** (inserted on page 12, line 158-169)

We examined the cross-correlation functions (CCFs) between the two-time series of common infectious disease count and COVID-19 disease count. We used the CCF to understand the time-lagged correlation between common infectious disease count and COVID-19 incidence. To compare the change in case numbers of common infectious diseases in 2020 with that in the previous 5 years (2015–2019), a difference-in-differences linear regression was applied for infectious diseases transmitted by droplets that are most susceptible to behavioral changes caused by COVID-19. The model included a categorical variable for each week, a categorical variable for the 2020 season (versus the 2015–2019 seasons) and the interaction variables for each week and the 2020 season, following the method described by Sakamoto et al. (2020) [5]. For the statistical analysis, Light Stone^®^ STATA^®^ ver.15 was used.

**Results** (inserted on page 18-19, line 291-299)

According to the difference-in-differences analysis, the activity of influenza was significantly lower since the second week in 2020 than during 2015-2019 (Fig 4). Similarly, respiratory syncytial virus was lower after 27 weeks, Group A streptococcal pharyngitis was lower after 10 weeks, hand, foot, and mouth disease was lower after 5 weeks, erythema infectiosum were lower after 13 weeks, herpangina was lower after 11 weeks, mumps was lower during 27 to 30 weeks, *Mycoplasma pneumoniae* pneumonia was lower after 25 weeks, pertussis was lower after 15 weeks, and rubella was lower after 31 weeks (Fig 4). However, legionellosis was more frequent throughout the year than in 2015–2019 (Fig 4).

**Figure legend** (page 19, line 301-303)

**Fig. 4 Difference-in-differences value in 2020 vs. that in 2015-2019 (95% credible interval for droplet transmitted disease).** Negative 95% credible interval indicates fewer cases in the 2020 than in the 5 previous years (*p*<0.05). Ctrl: credible interval

2) The authors have very thoughtfully extracted and categorized the reported case data. Is any data additionally available that could be used to examine the impact of reduced healthcare seeking or testing capacities on the conclusions? If possible, this should be investigated further. For example, is information available on changes in number of tests performed or in number of hospital admissions? If so, this could be used to perform a sensitivity analysis of the total 2020 case counts followed by the regression-based analysis described above (see Lee & Lin (2020) EID for an example). If this is not possible, then this potential issue should be discussed in more depth and earlier on in the manuscript (not just in the limitations and discussion of HIV/syphilis). For example, are there certain pathogens this will likely affect more than others in Japan? Might this be different in the sentinel vs. passive surveillance systems?

Reply to the reviewer: We showed the number of monthly hospital visitors of 2020 as Figure 5 with the number of monthly newly infected COVID-19 cases. Unfortunately, the number of outpatients for each disease cannot obtain from the national data. Therefore, we discussed the potential issue utilizing the data as follows:

**Discussion** (inserted to page 20-21, line331-354)

**Impact of refraining from physician visit for common infectious disease**

The spread of COVID-19 limited the clinical and laboratory diagnosis of common infectious diseases. In addition, people were unwilling to visit hospitals or clinics for diagnosis. Thus, the incidence of infectious diseases could be underreported. We showed the number of outpatients compared with that during the same month of the previous year at domestic medical clinics (Fig. 5). Outpatient numbers declined in May and September 2020 in all departments. Especially in pediatrics, the decrease is remarkable. Epidemic weeks 14–21 (April 7–May 25) of 2020 were the period during which the Japanese experienced the first state of emergency. According to mobile phone location analysis, the number of people in major cities in Japan was reduced by 40–60% during the state of emergency [20]. On average, the ratio of people who spent their time at home or in the neighborhood within a radius of 3 km from their homes had shown a decrease of over 50% during a long vacation (April 29 to May 6, 2020) under of state of emergency [21]. In other words, even the behavior of visiting hospitals and clinics may have been suppressed during this period. However, the decrease in outpatient numbers was temporary, and abstaining from visiting hospitals/clinics may have a minor impact. At least, for infectious diseases with low numbers of cases through 1 year, the effects of refraining from seeing a doctor may be considered to be negligible. Also, children who have a sudden fever or rash, such as measles or chickenpox, or patients who have high fever due to the flu may be less likely to refrain from seeing a doctor. It is often difficult for a citizen to distinguish COVID-19 from common infectious diseases. Therefore, even if individuals tended to abstain from visiting medical facilities, we suspect it had less effect on common infectious diseases that cause a high fever.

**Material and Methods** (inserted on page 11-12, line 149-156)

**Total number of outpatient cases in the medical clinics**

The total number of outpatient cases in the domestic medical clinics was obtained from the Ministry of Health, Labor and Welfare's estimated medical expenses database [11]. The “medical clinic” is meaning the places where doctors provide medical practice and do not have hospitalization facilities for patients or have hospitalization facilities for 19 or less patients. The number of cases referred to here is the number of medical fee statements (receipts), and each medical institution prepares one statement for one patient every month. The data was added as Supplemental Table S2.

**References** (Added a new reference cited in the above description)

11. Medical expenses data. The Ministry of Health, Labor and Welfare. [Cited 2021 August 31]. Available from: <https://www.mhlw.go.jp/bunya/iryouhoken/iryouhoken03/03.html> (see Table S2)

20. COVID-19 Mobility Trends Reports-Apple, Apple Maps. [Cited 2021 September 3]. Available from:

https://covid19.apple.com/mobility

21. Decrease rate of visitors in each prefecture during Golden Week. LocationMind xPop [Cited 2021

September 3]. Available from: https://locationmind.com/news/262/ (Japanese)

We added the explanation about the Fig 5 in the Results section.

**Results** (inserted on page 19, line 305-312)

**Relationship between the COVID-19 epidemic and total number of outpatient cases.**

The total number of outpatients is shown year-on-year in the same month (Fig 5, S2 Table). At the same time, the epidemic curves of COVID-19 were superimposed. The number of outpatients in any clinical departments decreased in May and September. The decrement was greatest in pediatrics. The transition was similar to the COVID-19 epidemic curve. We tried to calculate the cross-correlation coefficient between them, but could not analyze it for unknown reasons.

3) Why was the year 2019 alone used as the comparison for 2020? Why not compare several previous seasons, for example 2014-2019 with 2020, to account for some of the year-to-year variation?

Reply to reviewer: In Figure 2, the 2019 epidemic curve was used to show the connection from the previous year to the current disease epidemic. If the number of patients has decreased from the beginning of 2020 compared to the same month of the previous year, it is possible to know when it started last year　However, in Figure 3, we showed the average value from 2015 to 2019 as the comparison for 2020.

**Table legend of Fig 3** (page 17, line 267):

The blue thin lines (**-**) indicate the epidemic curves during 2015-2019.

Additional comments:

• In the abstract, it is unclear what methods and results the authors used to arrive at the conclusions. Additional details and using a structured abstract may help to improve clarity. Including the statistical analysis suggested above may also help in clarifying what the key findings are in the abstract.

Reply to reviewer: We included the statistical analysis suggested by reviewers in this study. Then, we revised the abstract as follows:

**Abstract** (inserted on page 2-3, line 34 to 45)

When the cross-correlation functions (CCFs) between the two-time series of common infectious disease count and COVID-19 disease count were examined, a strong correlation was shown for scrub typhus at rag -1 week (CCF 0.8705), herpangina at rag -15 week (CCF 0.6465). The overall infectious activity of droplet-transmitted diseases in 2020 was lower than the average of previous five years (2015–2019). According to the difference-in-differences analysis, the activity of influenza and rubella was significantly lower since the second week in 2020 than those in 2015–2019. Only legionellosis was more frequent throughout the year than that in 2015–2019. Lower activity was also observed in some contact transmitted, airborne-transmitted, and fecal-oral transmitted diseases. However, carbapenem-resistant Enterobacteriaceae, exanthema subitum, showed the same trend as the previous 5 years. In conclusion, our study has shown that public health interventions for the COVID-19 pandemic may have effectively prevented the transmission of most droplet-transmitted diseases and those transmitted through other routes.

• I don’t understand what the sentence on lines 68-69 is meant to indicate. Please rephrase or expand here.

Reply to reviewer: We revised the pointed out sentence as follows;

**Datasets about COVID-19** (page 4, line 68-71 in the first version**)**

Datasets on COVID-19 were extracted from the domestic infection status officially released by the Ministry of Health, Labour and Welfare of Japan based on the NESID [7]. The COVID-19 patients were diagnosed using polymerase chain reaction (PCR) or antigen testing for SARS-CoV-2.

↓

**Datasets about COVID-19** (page 4-5, line 78-86 in the revised version)

The COVID-19 pandemic from January 16, 2020, to December 31, 2020 was demonstrated by datasets from the National Epidemiological Surveillance of Infectious Diseases (NESID) under the Infectious Diseases Control Law. We obtained the open data about COVID-19 from January 16, 2020 to December 31, 2020 from the Ministry of Health, Labour and Welfare [7]. Then, data on the daily number of new positive cases who have taken polymerase chain reaction (PCR) for SARS-CoV-2 or antigen testing for SARS-CoV2 were used in this study. These domestic cases do not include cases of airport quarantine.

• In the methods, more details are needed on why the inclusion/exclusion criteria were applied. Why was 400 chosen as the minimum number of cases? Why were fulminant, invasive, and enteric infections other than rotavirus excluded?

Reply to the reviewer: In this paper, we intend to cover and report on many infectious diseases. However, it is also true that the number of cases of infectious diseases reflects that it is an important disease. Since there are 365 days in a year, we set the number to more than 400, considering more than one case per day. It is also true that the total number of invasive infections is small, but it is interpreted that invasive infections indicate the severity of the disease and do not necessarily reflect the frequency or route of infection. Therefore, we deleted those from this analysis.

**Material and Methods** (inserted on page 5, line 88-100)

**Datasets of common infectious diseas**e

Common infectious diseases were selected from the nationally notifiable diseases according to the following: i) we were excluded the diseases with l<400 cases of infection per year. Since there are 365 days in a year, we set the number to >400, considering more than one case per day. ii) We excluded fulminant and invasive infectious diseases, such as invasive pneumonia disease, invasive meningococcal disease, and severe invasive streptococcal disease. The total number of invasive infections is small; however, invasive infections indicate the severity of the disease and do not necessarily reflect the frequency or route of infection. Therefore, we deleted those from the analysis. iii) We excluded “infectious gastroenteritis”, which is a syndrome that induced by various causes such as bacteria, viruses, parasites. Difficulties arise when classifying it via transmission route. Therefore excluded. iv) In addition, we excluded monthly reports of infections, such as gonococcal infections or multi-drug-resistant *Pseudomonas aeruginosa* infection.

• I suggest using “common infectious diseases” rather than “representative infectious diseases” throughout the manuscript. If “representative” is meant to indicate something other than “common”, please explain further.

Reply to reviewer: We revised “representative infectious diseases” throughout the manuscript as follows:

Page 2 line 29, page 4 line 73, page 14 line 200, page 14 line 202, page 14 line 203, page 17 line 265

representative common infectious disease

↓

common infectious disease

• I suggest using the phrase “fecal-oral transmission” instead of “oral transmission”.

Reply to the reviewer: We revised according to your suggestion as follows

Page 9 line 137, page 9 line 138, page 11, page 28 line 500, page 29 line 502, page 29 line 504

oral transmission

↓

fecal-oral transmission

• In Figure 1, I suggest using the y-axis label “Number of cases per day”.

Reply to the reviewer: We revised according to your suggestion as follows:

The label of y-axis in Figure 1: Number of patients per day

↓

The label of y-axis in Figure 1: Number of cases per day

• In Figures 2-3, please use colorblind-friendly colors instead of green and red.

Reply to the reviewer: Thank you very much for your comment. We used gray, red and blue color with light gray background color in Fig 2. Similarly, we used a combination of blue and red in Fig 3.

My revision

We needed support for statistical analysis in this revision. Therefore, I added two authors for this paper.

Hiroyoshi Iwata, Clinical Pharmacology & Therapeutics, University of the Ryukyus School of Medicine

Shinichiro Ueda, Clinical Pharmacology & Therapeutics, University of the Ryukyus School of Medicine

We changed “Varicella” to “Chicken pox” because the name of “Chicken pox” is more common. Similarly, pertussis is sometimes used as the name of “Whooping cough”, so it is shown in parentheses where necessary.

In this revision, we added supplemental table and figure. Therefore, I described the supporting information.

**Supporting information** (inserted on page 47-48, line 858-878)

**S1 Fig. Cross-correlation graph.** S1A: Cross-correlation between COVID-19, and common　infectious diseases under the national sentinel surveillance. S1B: Cross-correlation between COVID-19, and common infectious diseases under the national notifiable disease surveillance. The X-axis indicates the correlation coefficient between COVID-19 and each common infectious disease. The Y-axis indicates Lag (week).

**S1 Table. Case numbers per week about COVID-19 and case number per week per sentinel cite of notifiable infectious diseases.** These weekly numbers of COVID-19 were calculated from the daily number of the new positive cases that have taken polymerase chain reaction for SARS-CoV-2 or antigen testing for SARS-CoV2. The data was obtained from the Ministry of Health, Labour and Welfare [7]. The numbers of notifiable infectious diseases were collected from the Infectious Diseases Weekly Reports in accordance with the National Epidemiological Surveillance of Infectious disease [8].

**S2 Table. The total number of out-of-hospital (outpatient and home medical care) cases in the medical clinics and insurance pharmacies compared with that in the same month of the previous year.** The number of cases referred to here is the number of medical fee statements (receipts), and each medical institution prepares one statement for one patient every month. The data is national data and are obtained from the Ministry of Health, Labor and Welfare's estimated medical expenses database [11].
