# Supplementary material for: Incidence of common infectious diseases in Japan during the COVID-19 pandemic
Source: PLoS One. 2022 Jan 12;17(1):e0261332. doi: 10.1371/journal.pone.0261332 (PMC8754328; doi:10.1371/journal.pone.0261332)
Supplement: Attachment — Submitted filename: Response_to_Reviewers_second revision.docx [file pone.0261332.s005.docx]

6. Review Comments to the Author

Please use the space provided to explain your answers to the questions above. You may also include additional comments for the author, including concerns about dual publication, research ethics, or publication ethics. (Please upload your review as an attachment if it exceeds 20,000 characters)

Reviewer #1: In general, the revision adequately addresses the issues I had raised in my initial review. I have only minor comments that might further be addressed:

**1. Incidence rather than activity, in the title?**

Reply to reviewer: I changed the word "activity" to "incidence" in the title according to the reviewer's suggestion as follows:

**Title page** (Corrected the first line of text on page 1)

Incidence of common infectious diseases in Japan during the COVID-19 pandemic

**2. Throughout the text, lag rather than rag, when referring to cross-correlations.**

Reply to reviewer: I duly changed the word "rag" to "lag" according to the reviewer’s comments.

**3. The authors might mention the impact of mumps vaccine. I suspect the authors’ comments concerning measles vaccine would also bear on mumps.**

Reply to reviewer: As the reviewer pointed out, it is true that there are populations that are susceptible to measles, rubella, and mumps. Therefore, the same discussion can be made for mumps and rubella. We duly revised the discussion about rubella and mumps as follows:

**Discussion** (revised on Page 25, Line 414 to Page 26, Line 431)

In the present study, rubella and HFMD showed a marked decrease throughout the year compared with 2015–2019. There may be several possible explanations for this. For example, in 2013, reported rubella cases were 14,344. However, a decline was noted in subsequent years, with 319 cases in 2014, 163 in 2015, 126 in 2016, and 93 in 2017 [30]. Since August 2018, there has been a rapid increase in rubella incidence: 2,941 in 2018 and 2,306 in 2019 [30]. The total number of rubella cases in 2020 was 100. This means that the epidemic may have been contained, and it is thought to have just returned to baseline, without the influence of COVID-19. In this study, the CCF of rubella showed a negative correlation coefficient over lag 0–lag 20 weeks, suggesting that it might be suppressed over a long period. Incidentally, vaccination is effective in preventing rubella. The Meals-Mumps-rubella (MMR) vaccine has been introduced in many countries worldwide. However, in Japan, the MMR vaccine has not been introduced due to side effects. In addition, there are generations with low vaccination rates for rubella (males born between 1962 and 1978). The rubella epidemic of 2018-19 mainly affected men in the indicated generations [30]. This suggests that Japan is still at risk for rubella epidemics in these susceptible populations. Therefore, the lower activity throughout the year may have been influenced by preventative behavioral changes associated with the COVID-19 epidemic.

**Discussion** (revised on Page 26, Line 439 to Line 449)

The present study showed that the mumps epidemic curve was stable. The patient count of mumps has been below 0.2 per sentinel site since 2018 and has been on a downward trend each year [8]. According to the present CCF analysis of mumps, the strongest negative correlation was obtained at lag 1 week. Vaccination against mumps has also become compulsory in many countries. However, the vaccination is still voluntary in Japan. According to the National Epidemiological Surveillance of Vaccine-Preventable Diseases, the vaccination coverage in recent years was 30~ 40% and the antibody coverage using sera stored in domestic serum banks was approximately 70% [32]. This means that there is a population that is susceptible to mumps in Japan. Therefore, the epidemic may have been controlled by the behavioral change in this susceptible population.

32. National Institute of Infectious Diseases. Mumps (infectious parotitis) in Japan, as of September 2016. IASR 2016;37: 185-186. Available from:

<https://www.niid.go.jp/niid/images/idsc/iasr/37/440e.pdf>

4. A methodological issue: the authors might mention in the methods what range of values was used when calculating moving averages (e.g., 5 point moving average?)

Reply to reviewer: The moving averages were calculated with 2 points. Therefore, we mentioned it in the materials and methods as follows;

.

**Material and Methods** (inserted on Page 12, Line 146 to Line 147)

For the epidemic curves of "COVID-19" and "common infectious diseases:, a moving average with 2 points was calculated.

5. One upshot of cross-correlation values is “interpretation” of the lag (or, range of lags) that leads to maximal or minimal cross-correlation values. E.g., one might speculate that periods of high COVID-19 incidence might in some sense tamp down or delay reported incidence of certain other diseases. Which diseases are or are not so affected would also be of interest.

Reply to reviewer: We have duly added an interpretation of the lag (or range of lags) in the materials and methods and revised the results and discussion of the cross-correlation function as follows:

**Material and Methods** (revised on Page 12, Line 147 to Line 154)

**Statistical analysis**

The cross-correlation function (CCF) was used to understand whether there was a time-lagged correlation between common infectious disease count and COVID-19 incidence. CCF is a function that expresses the similarity between two-time series and gives information about how similar and displaced one-time series is to the other. CCF takes a value in the range of -1 (negative correlation) to 1 (positive correlation). If the correlation value exceeds the confidence level, then the two series are correlated. The cross-correlation between the two variables is statistically significant at approximately the 5% level of significance.

**Result** (Page 17, Line 253 to Page 18, Line 265)

Additionally, we examined the CCF between the two-time series of COVID-19 and common infectious disease counts. The incidence of scrub typhus peaked significantly 1 week earlier than the third peak of COVID-19 incidence (correlated efficient=0.87). Herpangina showed a significant peak 15 weeks earlier than the second peak time of COVID-19 incidence (cross-correlation values=0.65). The strongest negative correlation (cross-correlation values=0.40) was obtained at lag 1 week for mumps. Influenza, group A Streptococcal pharyngitis, erythema infectiosum, epidemic keratoconjunctivitis, *Mycoplasma pneumoniae* pneumonia, infectious gastroenteritis (rotavirus), pertussis, rubella, measles showed a negative correlation with COVID-19 in the lag from minus 20 weeks to 0 weeks. Sexually transmitted diseases, including amoebiasis, AIDS, and syphilis, reached their lowest peaks 1 to 2 weeks later than the peak in COVID-19 incidence. A closer look at the epidemic curve showed a phenomenon with a slight deviation from each peak of COVID-19.

**Discussion** (inserted on Page 27, Line 460 to Line 463)

Keratoconjunctivitis showed a negative correlation against COVID-19 in the lag from minus 20 weeks to 0 weeks. This may suggest that the hand-washing and hand-disinfection practices triggered by the COVID-19 epidemic also had a long-term effect on reducing the prevalence of contact infections.

**Discussion** (inserted on Page 33, Line 594 to Page 34, Line 599)

In this study, we also examined the CCFs between the two-time series of scrub typhus and COVID-19 disease counts. The peak incidence of COVID-19 coincided with the peak incidence of scrub typhus exhibiting a 1-week lag. Although there is a large seasonal bias in the occurrence of scrub typhus disease, we cannot deny the possibility that the release from behavioral inhibition by COVID-19 epidemic led to an increase in the incidence of scrub typhus infection.

**Discussion** (inserted on Page 35, Line 635 to Page 36, Line 638)

The lowest peak of current incidents of sexual transmitted disease showed a lag of few weeks than the peak of COVID-19 by cross-correlation analysis. The results may support the possibility that people became fearful when they saw the rapid increase in the number of new COVID-19 cases and changed their behavior.

6. The difference in differences methodology makes some strong assumptions on the putative relationship between the two series. The authors might comment on the validity of these assumptions in their context.

Reply to reviewer: We duly added the comment on the validity of these assumptions in the text.

**In the section of Material and Methods** (inserted on Page 13, Line 161 to Line 166)

We made two assumptions for the difference-in-difference linear regression in our preliminary experiments. First, the parallel trend assumption was valid for both incidences because the current incidents and incidences of the previous 5 years of common infections were parallel. The common shock assumption was also valid, as it showed a similar change when an event (the epidemic of COVID-19) occurred, indicated the appropriateness of this study design.

Reviewer #2: The authors have very thoroughly addressed my comments. The difference-in-difference analysis helps to show a clear story of reduced reporting of infections transmitted by droplets in Japan in 2020. The analysis and discussion of changes in outpatient behavior is also very helpful for understanding the context of broader health seeking during the pandemic. I only have a few remaining minor points:

1) The cross-correlation results in the abstract lines 36 to 38 and results lines 256 to 257 are hard to follow. What does rag -1 week mean? I suggest using more common language there. Please also provide a definition for what the numbers provided in parentheses represent, and limit to 2 decimal places. In addition, what does “among them” on line 257 refer to if there were no correlations with COVID-19 as stated in the previous sentence (or perhaps that is a typo?). It would also be helpful to include a short explanation of what the implications/importance of this result is, and/or what the rationale was for performing this analysis.

Reply to reviewer: Apologies for mistakenly using the word "rag" for "lag". We duly revised the results of cross-correlation in the abstract lines 36 to 38 and the results lines 256 to 257. In the Summary and the Results, "CCF" has been reworded to cross-correlation values, and numbers in parentheses have been limited to two decimal places. We have also reviewed the overall description of cross-correlation functions and have then included an explanation of “lag” and the benefits that can be derived from cross-correlation functions in the Material and Methods.

**Abstract** (revised on Page 2, Line 33 to Line 36)

The cross-correlation functions between the two-time series of common infectious and COVID-19 disease counts were examined. Many droplet-borne diseases, infectious gastroenteritis (rotavirus), and measles showed a negative correlation against COVID-19 in the lag from minus 20 weeks to 0 weeks.

**Material and Methods** (inserted on Page 12, Line 147 to Line 154)
**Statistical Analyses**

The cross-correlation function (CCF) was used to understand whether there was a time-lagged correlation between common infectious disease count and COVID-19 incidence. CCF is a function that expresses the similarity between two time series, and gives information about how similar and displaced one time series is to the other. CCF takes a value in the range of -1 (negative correlation) to 1 (positive correlation). If the correlation value exceeds the confidence level, then the two series are correlated. The cross-correlation between the two variables is statistically significant at approximately 5% level of significance.

**Result** (revised on Page 17, Line 253 to Page 18, Line 265)

Additionally, we examined the CCF between the two-time series of COVID-19 and common infectious disease counts. The incidence of scrub typhus peaked significantly 1 week earlier than the third peak of COVID-19 incidence (cross-correlation values=0.87). Herpangina showed a significant peak 15 weeks earlier than the second peak time of COVID-19 incidence (cross-correlation values=0.65). The strongest negative correlation (cross-correlation values=0.40) was obtained at lag 1 week for the mumps. Influenza, group A Streptococcal pharyngitis, erythema infectiosum, epidemic keratoconjunctivitis, Mycoplasma pneumoniae pneumonia, infectious gastroenteritis (rotavirus), pertussis, rubella, measles showed a negative correlation against COVID-19 in the lag from minus 20 weeks to 0 weeks. Sexually transmitted diseases, including amoebiasis, AIDS, and syphilis, reached their lowest peaks 1 to 2 weeks later than the peak in COVID-19 incidence. A closer look at the epidemic curve showed a phenomenaon with a slight deviation from each peak of COVID-19.

2) I suggest removing the sentence in lines 311 to 312, unless the authors can identify the reason that the CCF analysis “didn’t work”.

Reply to reviewer: We duly deleted the sentence in page 19, lines 311 to 312 in the second submitted manuscript.

3) There are several instances in the text where it sounds like the authors are implying a causal link between COVID-19 measures and reductions in reported cases. For example lines 435 to 436: “Therefore, the COVID-19 control measures did not affect the spread of legionellosis.” The analysis in this study is descriptive, not causal, so this section could read something like: “COVID-19 control measures were not associated with changes in reported cases of legionellosis, which is consistent with the transmission route. etc.”. Other examples where this should be clarified include lines 426-427, 442-444, 500-501, 511-513. In addition, the titles of these sections (currently: “Effect of COVID-19 measures on the incidence...”) should be revised to read something like: “Associations between COVID-19 measures and incidence of XX” or “Patterns in incidence of XX pathogens during the 2020 COVID-19 pandemic”.

Reply to reviewer: In accordance with the reviewer's suggestion, we have removed all sentences that implied a causal relationship. The following text has been removed.

**Discussion** (Second submitted manuscript: Page 25, Line435-436)

~~Therefore, the COVID-19 control measures did not affect the spread of legionellosis.~~

**Discussion** (Second submitted manuscript: Page 25, Line426-427)

~~In the present study, we considered that preventive measures for COVID-19 suppressed the small epidemic of HFMD caused by coxsackievirus A16~~

**Discussion** (Second submitted manuscript: Page 26, Line442-444)

~~Therefore, we considered that behavior modification, such as hand hygiene, for preventing COVID-19 was also effective against epidemic keratoconjunctivitis.~~

**Discussion** (Second submitted manuscript: Page 28, Line 500-501)

~~Nevertheless, the suppression of the measles epidemic without any outbreaks demonstrates the effectiveness of public health interventions.~~

**Discussion** (Second submitted manuscript: Page 29, Line 511-513)

~~Nevertheless, the present results suggest that infectious gastroenteritis caused by rotavirus can be prevented by proper hand washing, disinfection, gargling, washing, and sterilization in household settings.~~

Reply to reviewer: In response to the reviewers' comments, we revised the title of each section have duly made the following amendments to each section.

**The title of each section**

Page22, Line 364 - Line 365: Associations between COVID-19 measures and the incidence of droplet-transmitted diseases

Page27, Line 455 - 456: Associations between COVID-19 measures and the incidence of contact-transmitted diseases

Page 28, Line 478 - 479: Associations between COVID-19 measures and the incidence of airborne transmitted diseases

Page 30, Line 525 - 526: Associations between COVID-19 measures and the incidence of fecal orally transmitted diseases

Page 33, Line 585 - 586: Associations between COVID-19 measures and the incidence of vector-borne diseases

Page 34, Line 601 -602: Associations between COVID-19 measures and the incidence of STDs

Author’s corrections: In line with this revision, the authors have duly made the following corrections to the discussion of measles/rotavirus infections.

**Discussion** (inserted on Page 30, Line 515 to Line 523)

~~The measles vaccine has been said to provide lifelong immunity; however, it is known that antibody levels decrease even after vaccination in the absence of recent epidemics [46]. For this reason, even in Japan, there are populations with low antibody levels, and small outbreaks can occur at any time.~~ The overall measles antibody prevalence in 2020 was 96.3%, although the antibody prevalence among children aged 1 year in 2020 was 69.8%, a significant decrease from 81.6% in 2019 [47]. This has been attributed to a temporary abstaining from vaccination [47]. However, the impact on the measles epidemic is thought to have been short-term and small. In addition, the estimated infected areas of the 12 measles cases reported in 2020 were 7 national, 4 overseas, and 1 unknown [47]. While the number of domestic cases has decreased, the proportion of imported cases is becoming more apparent. If there had been no entry restrictions due to the COVID-19 pandemic, such imported cases might have increased.

47) National Institute of Infectious Disease. Measles in Japan, as of June 2021. IASR. 42:177-182, 2021. Available from: <https://www.niid.go.jp/niid/en/typhi-m/iasr-reference/1687-iasr-backnomber-e.html>

**Discussion** (inserted on Page 31, Line 533 to Line 537)

Among fecal-oral infections, only rotavirus infection showed a negative correlation between lag 0 and 25 weeks in the CCFs analysis. This suggests that rotavirus infection generally occurs in the home rather than in eating establishments and that appropriate hygiene behavior in the home may have had a long-term effect.
