# Supplementary material for: Incidence of common infectious diseases in Japan during the COVID-19 pandemic
Source: PLoS One. 2022 Jan 12;17(1):e0261332. doi: 10.1371/journal.pone.0261332 (PMC8754328; doi:10.1371/journal.pone.0261332)
Supplement: Attachment — Submitted filename: PLOSONE_29nov21.doc [file pone.0261332.s006.doc]

**Proteogenomics Research Institute for Systems Medicine**

***Discovery through the Spectrum of Science***

505 Coast Boulevard South

La Jolla, California 92037	USA

29 November 2021

PLOS Editorial Board

The authors have made appropriate changes to their manuscript, reflecting suggestions from previous reviewers’ comments, and I would recommend publication of their work.

James Koziol
